# ID 282 - JAV (Jornada Assistencial de Valor) RARAS: análise de custos utilizando TDABC de pacientes com distrofia muscular de Duchenne na Policlínica Codajás/AM

**DOI:** 10.5327/2237-9622.2025.v34s1.282

**Published:** 2025-11-25

**Authors:** Myrianne Gilsara Soares e Barbosa, Camila Azevedo, Vânia Mesquita Gadelha Prazeres, Lorena Melo de Jesus, Andreia Batista Nogueira de Oliveira, Marcelo Nita, Luana Lopes, Thiago Godoy, Têmis Félix

**Keywords:** distrofia muscular de Duchenne, análise de custos, *Time-Driven Activity-Based Costing*

## Abstract

**Introdução:**

O Estudo JAV-RARAS integra o projeto da Rede Nacional de Doenças Raras (RARAS), cujo propósito é compreender e quantificar a Jornada Assistencial de Valor (JAV) dos indivíduos que sofrem de 21 doenças raras no Brasil. O objetivo é estabelecer registros das patologias raras, definir parâmetros de valor para os pacientes e avaliar a relação custo-efetividade, buscando otimizar a distribuição de recursos em diversas regiões do País. Este estudo tem como objetivo específico mensurar e descrever os custos associados à jornada assistencial de pacientes com distrofia muscular de Duchenne na Policlínica Codajás/AM, como parte do projeto JAV-RARAS, um componente da Rede Nacional de Doenças Raras no Brasil, aplicando a metodologia (TDABC).

**Método:**

O TDABC envolve a utilização de dados referentes a diagnósticos, tratamentos e acompanhamentos no contexto do estudo JAV-RARAS. Inicialmente, foi realizado o mapeamento da jornada de cuidados em centros participantes do estudo para 21 doenças raras, quantificando o tempo e os recursos associados às etapas de diagnóstico, tratamento e acompanhamento. Os custos diretos foram obtidos por meio de entrevistas e registros administrativos de profissionais de enfermagem e médicos na Policlínica Codajás, localizada no Amazonas. Além da análise dos custos diretos, foram identificadas as atividades na jornada do paciente com DMD, a alocação de recursos, o custo por unidade e a mensuração do tempo de duração das atividades.

**Resultados:**

O custo médio anual para o tratamento de um paciente com distrofia muscular de Duchenne é de R$ 397.241,34, sendo a maior parte destinada às despesas com medicamentos. A distribuição dos custos compreende recursos humanos (R$ 6815,70), medicamentos (R$ 389.622,84) e exames (R$ 802,56). Quanto à origem dos recursos, aproximadamente 1,77% provem da Policlínica Codajás, enquanto 0,64% é financiado pelo Sistema Único de Saúde (SUS) em outras localidades, e 97,5% dos recursos são acessados por meio da judicialização de medicamentos.

**Conclusão:**

Este estudo ressalta a importância de analisar e mapear os custos e o acesso a intervenções terapêuticas no tratamento da distrofia muscular de Duchenne. A maior parte dos gastos anuais está associada aos medicamentos, destacando a necessidade de uma compreensão aprofundada dos fatores que influenciam esses custos, sobretudo no que diz respeito à judicialização dos medicamentos. A exploração de alternativas terapêuticas mais eficazes tem o potencial de reduzir os custos em longo prazo e aprimorar os desfechos clínicos dos pacientes.

